# QuickStats

**Published:** 2014-03-14

**Authors:** FROM THE NATIONAL CENTER FOR HEALTH STATISTICS

**Figure:**
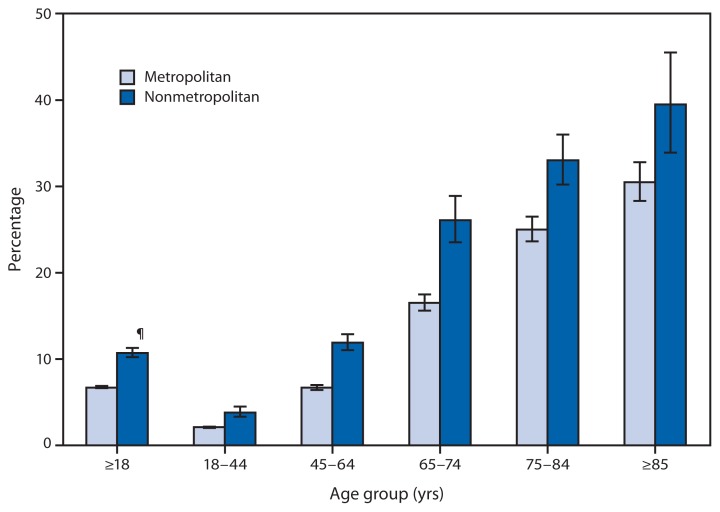
Percentage of Adults Aged ≥18 Years Who Have Lost All Their Natural Teeth,* by Age Group and Type of Locality^†^ — National Health Interview Survey, United States, 2010–2012^§^ * Based on response to the question, “Have you lost all of your upper and lower natural (permanent) teeth?” ^†^ The designation of a place of residence as metropolitan or nonmetropolitan is determined by whether the household resides within a metropolitan statistical area, defined as a county or group of contiguous counties that contains at least one urbanized area of ≥50,000 population. Surrounding counties with strong economic ties to the urbanized area are also included. Nonmetropolitan areas do not include a large urbanized area and are generally thought of as more rural. ^§^ Estimates are based on household interviews of a sample of the civilian, noninstitutionalized U.S. population and are derived from the National Health Interview Survey sample adult component. Estimates for the ≥18 years age category are calculated using age-specific percentages for five age groups: 18–44, 45–64, 65–74, 75–84, and ≥85 years. ^¶^ 95% confidence interval.

During 2010–2012, the percentage of adults aged ≥18 years who had no natural teeth was higher in nonmetropolitan areas than in metropolitan areas for all age groups. The percentage of adults with no natural teeth also increased steadily with age in metropolitan and nonmetropolitan locations. Among persons aged ≥85 years in nonmetropolitan locations, 40% had lost all their natural teeth, compared with 31% of those in metropolitan areas. Among adults aged 18–44 years, the percentages were 3.8% in nonmetropolitan areas and 2.1% in metropolitan areas.

**Sources:** National Health Interview Survey, 2010–2012. Available at http://www.cdc.gov/nchs/nhis.htm.

CDC. Health Data Interactive. Available at http://www.cdc.gov/nchs/hdi.htm.

**Reported by:** Ellen A. Kramarow, PhD, ekramarow@cdc.gov, 301-458-4325.

